# Chinese Sexual Minority Male Adolescents’ Suicidality and Body Mass Index

**DOI:** 10.3390/ijerph15112558

**Published:** 2018-11-15

**Authors:** Yeen Huang, Pengsheng Li, Zhisheng Lai, Xiaofei Jia, Di Xiao, Tian Wang, Lan Guo, Ciyong Lu

**Affiliations:** 1 Department of Medical Statistics and Epidemiology, School of Public Health, Sun Yat-sen University, Guangzhou 510080, China; huangyen@mail2.sysu.edu.cn (Y.H.); superplant@163.com (P.L.); Xiaodi@mail2.sysu.edu.cn (D.X.); wangt97@mail2.sysu.edu.cn (T.W.); guolan3@mail.sysu.edu.cn (L.G.); 2 Yuexiu District Center for Disease Control and Prevention, Guangzhou 510080, China; laizhisheng2018@163.com; 3 Tianhe District Center for Disease Control and Prevention, Guangzhou 510655, China; jiaxiaofei2018@126.com; 4 Guangdong Provincial Key Laboratory of Food, Nutrition and Health, Sun Yat-sen University, Guangzhou 510080, China

**Keywords:** sexual minority, suicide, adolescents, body mass index, moderating effect

## Abstract

Excess weight status may increase the risk of suicidality among sexual minority females, but few studies have examined this suicidality disparity in sexual minority males. This study examined the association between sexual minority status and suicide attempts in Chinese male adolescents and tested whether body mass index (BMI) had a moderating effect on that association. Data were collected from 7th to 12th graders from seven randomly selected provinces of China in the 2015 School-Based Chinese Adolescents Health Survey. In total, 72,409 male students completed the questionnaires regarding sexual attraction, self-reported weight and height, and suicide attempts. After adjustment for covariates, sexual minority status was associated with suicide attempts among male students (AOR = 1.74, 95% CI = 1.57–1.93). Stratification analyses showed that BMI category moderated this association; compared with the results before stratification analyses, sexual minority males who were obese had increased risk of suicide attempts (AOR = 2.15, 95% CI = 1.09–4.24), sexual minority males who were overweight had reduced odds of suicide attempts (AOR = 1.40, 95% CI = 1.01–1.92), and no significant association change was found in sexual minority males who were underweight (AOR = 1.82, 95% CI = 1.43–2.33). Our study indicated that BMI moderated the risk of suicide attempts in sexual minority males. Suicide prevention targeting sexual minority males should be focused on weight status disparity and the creation of a positive climate to reduce minority stressors due to body image.

## 1. Introduction

Suicide is a leading cause of death among adolescents and youth adults, and suicide attempts are an important marker of suicidality [1,2]. A previous study showed that suicide attempts are currently a serious problem among adolescents in developing countries [3]. China is the largest developing country, with a high rate of suicidality, and the suicide patterns stratified by sex in China are different from those in Western countries [4,5]. Previously, in China, females had a consistently higher risk of suicide attempts than males [5]; however, this pattern reversed in 2008 [6]. Therefore, because male adolescents may be at increased risk of suicide attempts in China, an understanding of the risk factors and causes of suicide attempts is imperative to prevent suicidality among this group.

Sexual minority status (e.g., individuals who experience same-sex attraction, engage in same-sex sexual behavior, or self-identify as gay, lesbian, or bisexual) is a well-known risk factor for suicide attempts in male adolescents due to their difficulties in dealing with minority stressors [7]. A previous health survey of adolescents from the United States reported that 5.0–28.1% of sexual minority males had past-year suicide attempts and that the rate of suicide attempts was nearly two to seven times higher for sexual minority males than for heterosexual males [8,9]. It is well known that the cultural background of Western countries is different from that of Asian countries, especially China. Research in China showed that 4.6–12.6% of sexual minority males had a lifetime prevalence of suicide attempts, which was a proportion that was approximately 5.8 times higher than that in the general male population [10,11]. There is evidence of a strong association between sexual minority status and suicide attempts in Chinese male adolescents.

It is well-known that physical abuse and school victimization may increase the likelihood of suicidality for sexual minority adolescents [12], and anti-discrimination policy will contribute to the prevention of their suicide attempts [13]. Moreover, overweight (AOR = 1.40, 95% CI = 1.32–1.49) and obesity (AOR = 1.91, 95% CI = 1.71–2.14) have been showed to be important reasons for adolescents to be bullied at school [14]. Therefore, weight status might associate with suicidality for sexual minority adolescents. Previous research has showed a clear relationship between weight status and suicide attempts among male adolescents [15]; however, whether this association also exits in sexual minority males and how does weight status affect suicide attempts in this group are still unclear. One previous longitudinal study demonstrated that sexual orientation may be associated with body mass index (BMI) among male adolescents [16] and that sexual minority males had higher BMI in early adolescence than their heterosexual peers [17]. Moreover, a prior study in Dutch adolescents reported that obese boys were more likely to have lifetime suicide attempts than their normal weight peers [15]. Two large-scale cohort studies found an inverse association between BMI and suicide attempts among male adults [18,19]. Therefore, BMI may play a moderating role in the association between sexual minority status and suicide attempts among male adolescents. However, no previous study has examined this moderating role. Therefore, the objectives of this study were to estimate the association between sexual minority status and suicide attempts in Chinese male adolescents and to test whether BMI had a moderating effect on this association.

## 2. Materials and Methods

### 2.1. Study Design and Participants

We utilized data from the 2015 School-Based Chinese Adolescents Health Survey (SCAHS) [20,21], an ongoing, large-scale health-related behavior survey among Chinese adolescents (grades 7–12). The SCAHS has been conducted every two years since 2007, and the 2015 survey was the most recent version conducted in seven Chinese provinces [22]. In the 2015 SCAHS, students were selected via a 4-stage, stratified-cluster, random-sampling method. In total, 150,822 students completed the questionnaires (response rate of 95.9%), and 72,409 of those students were males. Therefore, in total, 72,409 male students were included in the analysis.

### 2.2. Data Collection

All students from the chosen classes were given a standardized self-administered questionnaire on the day of the survey to complete in the classroom during a normal class period (40 or 45 min). To protect students’ privacy, the questionnaire was completed by each student participant anonymously under the guidance of investigators and without the presence of teachers or other school personnel (to avoid any potential information bias). After collecting the questionnaires, investigators checked them carefully and contacted the students in a timely manner when they found important missing data. Investigators received standardized training by the research team, and quality control was implemented during data collection. All data were collected from November 2014 to January 2015.

### 2.3. Ethical Statement

This study was conducted in accordance with the Declaration of Helsinki, and was approved by the Institutional Review Board (ethic code: L2014063) of School of Public Health, Sun Yat-sen University. Written informed consent was obtained from each participating student who was at least 18 years old or from one of the student’s parents (or legal guardians) if the student was under 18 years old.

### 2.4. Measures

#### 2.4.1. Sexual Minority Status

Sexual minority status was measured by asking students the following question regarding sexual attraction: “In a romantic relationship, which kind of person are you attracted to?” Responses options included the following: ‘opposite sex’, ‘same sex’, ‘equally opposite sex and same sex’, and ‘unsure’ [9,23,24]. Students who reported ‘same sex’, ‘equally opposite sex and same sex’, or ‘unsure’ attraction were classified as sexual minorities. A similar question has been used in other studies of Chinese adolescents [24,25].

#### 2.4.2. Suicide Attempts

Suicide attempts were measured by asking the question “During the past 12 months, how many times did you actually attempt suicide?” and the students were asked to rate on a scale of zero or once or more [26,27].

#### 2.4.3. Body Mass Index Category

BMI (kg/m^2^) was used as a measure of relative body weight and was calculated from self-reported weight (kg) and height (m) [28,29]; self-reported weight and height and the BMI calculated from these values have been showed to correlate strongly and reliably with measured values [30] and are reliable for predicting obesity status in adolescents [31,32]. Students were classified as underweight, normal weight, overweight, and obesity based on their BMI, and the classification criteria were as follows:

For male students ≤18 years, underweight was defined as a BMI ≤the referent age- and sex-specific percentiles in the national screening standard for underweight in Chinese school-aged children and adolescents (WS/T456-2014) [33]; normal weight was defined as a BMI greater than the referent age- and sex-specific percentiles of underweight and less than the referent age- and sex-specific percentiles of overweight; overweight was defined as a BMI ≥the referent age- and sex-specific 85th percentile but less than the 95th percentile according to the reference for adolescents developed by the Working Group on Obesity in China (WGOC) [34]; obesity was defined as a BMI ≥the referent age- and sex-specific 95th percentile according to the reference for adolescents developed by the WGOC. For male adolescents >18 years, the cut-off points for BMI for underweight, overweight, and obese were 18.5, 24, and 28, respectively, according to the reference for adults developed by the WGOC [35,36].

#### 2.4.4. Covariates

Covariates included age, academic pressure, cigarette smoking, alcohol consumption, school victimization, and depressive symptoms, which have previously been reported to be associated with suicidality among sexual minority adolescents [8,9,37,38].

Academic pressure was assessed by asking about the student’s personal appraisal of academic stress of the school year. The responses included “none”, “less”, and “medium or great”. Cigarette smoking was measured by asking the following question: “During the past 30 days, on how many days did you smoke cigarettes?” Students who selected answers indicating 1 or more days were classified as smokers [39]. Alcohol consumption was assessed with the following question: “During the past 30 days, on how many days did you drink alcohol?” Students who selected answers indicating 1 or more days were classified as alcohol consumers [40]. School victimization was assessed according to the definition of bullying from the Olweus Bully/Victim Questionnaire [41]; students were asked the following question: “Have you been bulled at school in the previous month?” The responses included “no”, “1–2 days”, “3–5 days”, “6–9 days”, “1–19 days”, and “more than 20 days”. Students who reported being bulled with a frequency of 3 days or more were classified as having experienced school victimization [25,42]. The Chinese version of the Depression Self-Rating Scale for Children (DSRSC) was used to identify whether individuals had depressive symptoms [43,44]. The score range of the DSRSC was 0-54, and scores greater than 15 points indicated depressive symptoms. This scale has been demonstrated to be valid and reliable in the Chinese adolescent population.

### 2.5. Statistical Analysis

First, descriptive analyses were conducted to describe demographic characteristics, prevalence of BMI categories, and suicide attempts in sexual minority males and heterosexual males; Rao-Scott *χ*^2^ tests were used to compare the differences between groups. Second, univariate logistic regression models were performed to explore the association between sexual minority status and suicide attempts among male adolescents. The variables that were significant at the 0.10 level in univariate analyses or that had been widely reported in previous studies were simultaneously included in the multivariate logistic regression models to evaluate the independent association between sexual minority status and suicide attempts. Odds ratios (OR) and 95% confidence intervals (CI) were obtained from the logistic regression models. Third, to investigate whether the association between sexual minority status and suicide attempts was moderated by BMI category, we tested the interaction between sexual minority status and BMI category. If the interaction was significantly associated with suicide attempts, we performed stratification analyses to measure the association between sexual minority status and suicide attempts in male adolescents with normal weight, underweight, overweight, and obesity. Missing data accounted for less than 3.1% of all relevant variables, and observations with missing data were eliminated in the statistical analyses. In prevalence estimates and logistic regression analyses, appropriate sampling weights and estimation procedures that accounted for the complex sampling design were used. All statistical analyses were conducted using SAS 9.3 (SAS Institute, Inc., Cary, NC, USA). A two-tailed *p*-value of less than 0.05 was considered statistically significant.

## 3. Results

### 3.1. Characteristics of Participants and the Prevalence of BMI Categories and Suicide Attempts

As shown in Table 1, of the 72,409 male students analyzed, 80.1% self-reported as heterosexuals and 19.9% self-reported as sexual minorities (same-sex attraction: 1.4%, both-sex attraction: 1.9%, and unsure: 16.6%). The male students ranged in age from 12 to 20 years, and the proportion of male students who ranged in age from 12 to 13 and from 14 to 15 years was higher among sexual minority males than heterosexual males (*p* < 0.001). Compared with their heterosexual peers, sexual minority males were more likely to report school victimization and depressive symptoms (*p* < 0.001).

The weighted prevalence of having past-year suicide attempts was significantly greater in sexual minority males than in heterosexual males (4.2% vs. 2.2%, *p* < 0.001). Compared with their heterosexual peers, sexual minority males were more likely to fall outside of the normative BMI categories (*p* < 0.001).

### 3.2. Association of Sexual Minority Status, BMI Category, and Their Interaction with Suicide Attempts

In the unadjusted models (Model 1 in Table 2), sexual minority status, BMI category, and their interaction were significantly associated with past-year suicide attempts (*p* < 0.05). Furthermore, after adjustment for age, academic pressure, cigarette smoking, alcohol consumption, school victimization, and depressive symptoms (Model 2 in Table 2), the above associations remained statistically significant (*p* < 0.05). Sexual minority status was associated with past-year suicide attempts (adjusted odds ratios (AOR) = 1.74, 95% CI = 1.57–1.93). Male students with obesity (AOR = 1.35, 95% CI = 1.11–1.64) were more likely to have past-year suicide attempts than those with normal weight, underweight, or overweight. The interaction of sexual minority status and BMI category was statistically significant in the adjusted models for past-year suicide attempts (*p*-interaction = 0.025).

### 3.3. Association between Sexual Minority Status and Suicide Attempts Stratified by BMI Category

Stratification analyses were conducted separately for male students with normal weight, underweight, overweight, and obesity (Table 3). After stratification analyses, the association between sexual minority status and past-year suicide attempts was moderated by BMI category; compared with the results before stratification analyses, sexual minority males who were obese had a greater risk of past-year suicide attempts (AOR = 2.15, 95% CI = 1.09–4.24), sexual minority males who were overweight had reduced odds of past-year suicide attempts (AOR = 1.40, 95% CI = 1.01–1.92), and no significant association change was found in sexual minority males who were underweight (AOR = 1.82, 95% CI = 1.43–2.33).

## 4. Discussion

Using data from the 2015 SCAHS, we found that BMI moderated the risk of suicide attempts in Chinese sexual minority males, and those with obesity had increased odds of suicide attempts. To our knowledge, this is the first nationally large-scale study to explore the potential moderating effect of BMI on the relationship between sexual minority status and suicide attempts among male adolescents. 

Consistent with previous studies [8,9], our results demonstrated that sexual minority males experienced a higher prevalence of past-year suicide attempts than their heterosexual peers. Moreover, after adjustment for covariates, sexual minority males still had a higher risk of past-year suicide attempts than their heterosexual peers. These results might be related to sexual minority stress associated with same-sex orientation [7]. Our findings support those of previous studies showing a ubiquitously elevated suicidality risk in sexual minorities, and suggest that Chinese sexual minority males remain particularly vulnerable to suicide attempts in comparison with their heterosexual peers.

Furthermore, previous studies found that BMI was an important factor influencing suicide attempts among males [18,45]. Similarly, our study demonstrated that there was a significant association between BMI category and past-year suicide attempts in Chinese male adolescents. Individuals with obesity were at greater risk for past-year suicide attempts than those with normal weight, underweight, or overweight. More importantly, our stratification analyses were conducted separately for normal weight, underweight, overweight, and obese male adolescents, and the results illustrated that the risk of past-year suicide attempts was increased in sexual minority males who were obese and was reduced in those who were overweight compared with the results before stratification analyses, suggesting that BMI category might have a moderating effect on the association between sexual minority status and suicide attempts. A prior study based on the Youth Risk Behavior Survey (YRBS) in the United States showed that sexual minority females who perceived themselves to be overweight had greater odds of past-year suicide attempts than all other sexual minority or perceived weight groups [46]. Moreover, previous cohort studies have showed that higher BMI is associated with a lower risk of suicide in male adults [18,19]. In contrast to previous findings, our results showed that sexual minority males might have a unique suicide pattern moderated by their weight status, which was different from that of sexual minority females and heterosexual males. Similar to previous studies which showed school victimization [47], anti-discrimination policy [13] and Gay-Straight Alliance [48] targeted sexual minority adolescents might have mediating or moderating effects on suicide risk in sexual minorities. Our study showed that weight status might also be one of the important influence factors of suicidality in sexual minority males, excess weight status were associated with higher risk of suicide attempts.

The mechanisms underlying suicidal behavior in sexual minorities are not entirely clear, and the increased suicide risk in obese sexual minority males might be related to their doubled level of minority stressors due to their sexual minority status and their excess weight status. The minority stress theory suggests that individuals may experience multiple minority stressors (e.g., prejudice and violence) if they are defined by stigmatized characteristics such as those of sexual minorities [7], overweight or obese people [49]. Adolescents who are sexually attracted to the same sex and are identified as obese may experience ridicule and are at substantial risk for bullying victimization; victimization in school environments is associated with negative individual outcomes such as depression and suicidal behavior [50]. In addition, our study also found that sexual minority males who were overweight had reduced risk of suicide attempts compared with those with normal weight or underweight. One possible explanation is that sexual minority males may desire to be thinner or more muscular than their heterosexual peers and show greater body dissatisfaction [51] and eating disorder symptomatology [52,53]. Such negative attitudes and behavior toward body image may cause sexual minority males to be more vulnerable to psychological and physical health issues (e.g., suicidal behavior) [54] even if their body image matches normative standards (e.g., weight status is in the normal range or underweight) of their current social environment. Therefore, sexual minority males might be a minority group with a unique suicide risk that is moderated by their weight status. Further studies are needed to understand the potential mechanisms underlying the association between sexual minority status and suicidality and to test whether weight status or other influential factors could strengthen or weaken these associations, such that suicidality intervention and prevention efforts could be more effectively targeted to these high-risk individuals. 

Based on our results, the following interventions to prevent suicidality among Chinese sexual minority males are recommended. First, schools and related public health organizations should create a friendly environment and atmosphere to reduce minority stressors against sexual minority males and those with obesity (e.g., supportive and positive regard from classmates and teachers). Second, families and communities should help sexual minority males accept their sexual orientation [55] and body appearance [56] to reduce pressures from sexual minority stressors (e.g., internalized homophobia) and body dissatisfaction (e.g., not comparing oneself unfavorably to another on the basis of appearance). Third, effective prevention and treatment of obesity should also be focused on to help sexual minority males reduce the physical and psychological issues related to suicidality.

Several limitations of our study should be noted when interpreting the results. First, the cross-sectional design of this study limited our ability to make causal inferences. Second, our study used a structured self-reported questionnaire to collect data, and we could not completely rule out the possibility of recall bias. Third, in our national large-scale study, the proportion of male students who responded “unsure” (16.6%) to the sexual minority status question was greater than in previous studies; those “unsure” students probably included not only individuals who were still unsure of their sexual orientation but also heterosexual students who were unwilling to disclose their sexual orientation or did not understand the question because of their younger age, which might result in an underestimation of the suicidality disparities in our study. Fourth, our study sample included students attending school but did not include adolescents who had dropped out of school or were absent from school on the day the survey was administered; suicidality may be more common among students who were absent, and this might lead to an underestimation of the suicidality disparities. Despite these limitations, the primary strengths of our study were that it included a nationally large-scale sample of Chinese male adolescents, which provided sufficient statistical power especially for the stratification analyses, and a representative sample could avoid the oversampling of sexual minorities. Moreover, our study is the first to explore the moderating effect of BMI on the association between sexual minority status and suicide attempts among male adolescents.

## 5. Conclusions

In China, obese individuals would suffer more stress from cultural preferences around the aesthetics and health of slim body, and such negative pressure might strengthen their association with suicidality especially for obese sexual minorities. Using a nationally large-scale sample of Chinese adolescents, our study indicated that BMI moderated the association between sexual minority status and suicide attempts in male adolescents, and this association was increased in sexual minority males who were obese and was reduced in those with overweight. These findings suggest that suicide prevention efforts targeting sexual minority males should be made, with close attention to disparity in weight status and the promotion of a positive climate to reduce minority stressors due to body image.

## Figures and Tables

**Table 1:** Sample characteristics and prevalence of BMI categories and suicide attempts, stratified by sexual minority status (*N* = 72,409).

Variables	Total No. (%)	Heterosexual Males No. (%)	Sexual Minority Males ^a^ No. (%)	*χ* ^2^	*p*-Value
Total	72,409 (100.0)	57,343 (80.1)	15,066 (19.9)		
Sexual minority status				NA	NA
Heterosexuals	57,343 (80.1)	NA	NA		
Sexual minorities males ^a^	15,066 (19.9)	NA	NA		
Same-sex attraction	NA	NA	1038 (1.4)		
Both-sex attraction	NA	NA	1445 (1.9)		
Unsure	NA	NA	12,583 (16.6)		
Age (years)				3891.72	<0.001
12–13	16,614 (23.1)	10,675 (19.0)	5939 (39.5)		
14–15	25,487 (35.5)	19,846 (34.9)	5641 (37.7)		
16–17	22,886 (30.1)	20,113 (33.3)	2773 (17.6)		
18–20	7422 (11.3)	6709 (12.8)	713 (5.2)		
Academic pressure				276.77	<0.001
None	12,795 (17.8)	9657 (16.9)	3138 (21.2)		
Less	31,765 (44.7)	24,811 (44.3)	6954 (46.3)		
Medium or great	27,849 (37.5)	22,875 (38.8)	4974 (32.5)		
Cigarette smoking				266.38	<0.001
No	65,407 (90.4)	51,271 (89.5)	14,136 (93.9)		
Yes	7002 (9.6)	6072 (10.5)	930 (6.1)		
Alcohol consumption				448.79	<0.001
No	56,957 (78.9)	44,158 (77.4)	12,799 (85.1)		
Yes	15,452 (21.1)	13,185 (22.6)	2267 (14.9)		
School victimization				74.24	<0.001
No	64,117 (88.5)	51,076 (89.0)	13,041 (86.2)		
Yes	8292 (11.5)	6267 (11.0)	2025 (13.8)		
Depressive symptoms				349.88	<0.001
No	58,682 (81.8)	47,273 (83.3)	11,409 (76.1)		
Yes	13,727 (18.2)	10,070 (16.7)	3657 (23.9)		
Suicide attempts				173.13	<0.001
No	70,404 (97.4)	55,991 (97.8)	14,413 (95.8)		
Yes	2005 (2.6)	1352 (2.2)	653 (4.2)		
BMI category				34.22	<0.001
Normal weight	49,995 (69.2)	39,879 (69.7)	10,116 (67.1)		
Underweight	11,130 (16.0)	8672 (15.8)	2458 (16.9)		
Overweight	7661 (10.2)	5998 (10.0)	1663 (10.8)		
Obesity	3623 (4.6)	2794 (4.5)	829 (5.2)		

Abbreviations: BMI, body mass index; No., number; NA, not applicable or no data available. ^a^ Sexual minority males included male adolescents who reported same-sex, both-sexes or unsure attraction. All numbers are unweighted, whereas all percentages are adjusted for sampling weights.

**Table 2:** Association of sexual minority status, BMI category, and their interaction with suicide attempts among male adolescents (N = 72,409).

Variable	Model 1		Model 2	
	Suicide Attempts		Suicide Attempts	
	OR (95% CI)	*p*-Value	AOR (95% CI)	*p*-Value
Sexual minority status				
Heterosexual males	1.00		1.00	
Sexual minority males ^a^	1.88 (1.71–2.06)	<0.001	1.74 (1.57–1.93)	<0.001
BMI category				
Normal weight	1.00		1.00	
Underweight	1.17 (1.03–1.32)	0.013	1.09 (0.96–1.23)	0.194
Overweight	1.06 (0.91–1.22)	0.457	1.07 (0.92–1.25)	0.383
Obesity	1.33 (1.10–1.60)	0.003	1.35 (1.11–1.64)	0.003
Sexual minority status × BMI category	-	<0.001	-	0.025

Abbreviations: BMI, body mass index; OR, odds ratios; AOR, adjusted odds ratios; CI, confidence intervals. ^a^ Sexual minority males included male adolescents who reported same-sex, both-sexes or unsure attraction. Model 1 for suicide attempts was unadjusted. Model 2 for suicide attempts was adjusted for age, academic pressure, cigarette smoking, alcohol consumption, school victimization, and depressive symptoms. Sexual minority status × BMI category: Interaction between sexual minority status and BMI category.

**Table 3:** Association between sexual minority status and suicide attempts among male adolescents, stratified by BMI category (N = 72,409).

Variable	Normal Weight	Underweight	Overweight	Obesity
	Suicide Attempts	Suicide Attempts	Suicide Attempts	Suicide Attempts
	AOR (95% CI)	AOR (95% CI)	AOR (95% CI)	AOR (95% CI)
Sexual minority status				
Heterosexual males	1.00	1.00	1.00	1.00
Sexual minority males ^a^	1.78 (1.56–2.02) ***	1.82 (1.43–2.33) ***	1.40 (1.01–1.92) ***	2.15 (1.09–4.24) ***

Abbreviations: BMI, body mass index; AOR, adjusted odds ratios; CI, confidence intervals. ^a^ Sexual minority males included male adolescents who reported same-sex, both-sexes or unsure attraction. All the models were adjusted for age, academic pressure, cigarette smoking, alcohol consumption, school victimization, and depressive symptoms. *** *p* < 0.001.
